# Ixabepilone-associated peripheral neuropathy: data from across the phase II and III clinical trials

**DOI:** 10.1007/s00520-012-1384-0

**Published:** 2012-03-02

**Authors:** Linda T. Vahdat, Eva S. Thomas, Henri H. Roché, Gabriel N. Hortobagyi, Joseph A. Sparano, Louise Yelle, Monica N. Fornier, Miguel Martín, Craig A. Bunnell, Pralay Mukhopadhyay, Ronald A. Peck, Edith A. Perez

**Affiliations:** 1 Weill Cornell Breast Center, Weill Cornell Medical College, New York, NY USA; 2 Kaiser Permanente, Oakland, CA USA; 3 Institut Claudius Regaud, Toulouse, France; 4 The University of Texas MD Anderson Cancer Center, Houston, TX USA; 5 Albert Einstein Comprehensive Cancer Center, Bronx, NY USA; 6 Centre Hospitalier de l’Université de Montréal—Höpital Notre Dame, Montreal, QC Canada; 7 Memorial Sloan-Kettering Cancer Center, New York, NY USA; 8 Servicio de Oncología Médica, Hospital Gregorio Marañón, Madrid, Spain; 9 Dana-Farber Cancer Institute, Boston, MA USA; 10 Bristol-Myers Squibb, Wallingford, CT USA; 11 Mayo Clinic, Jacksonville, FL USA

**Keywords:** Neuropathy, Ixabepilone, Epothilone, Breast cancer, Microtubules

## Abstract

**Purpose:**

Dose-limiting neuropathy is a major adverse event associated with most of the microtubule-stabilizing agent-based chemotherapy regimens. Ixabepilone, a semisynthetic analogue of the natural epothilone B, has activity against a wide range of tumor types. Peripheral neuropathy (PN), associated with ixabepilone treatment, is usually mild to moderate, predominantly sensory and cumulative. Preclinical studies demonstrate that ixabepilone and taxanes produce a similar neurotoxicity profile.

**Methods:**

We searched databases of phase II/III clinical trials involving patients receiving ixabepilone as a monotherapy or in combination with capecitabine for incidences of neuropathy. Potential risk factors for grade 3/4 PN were identified by a Cox regression analysis on a dataset of 1,540 patients with different tumor types across multiple studies.

**Results:**

Rates for incidence of ixabepilone-induced severe PN (Common Terminology Criteria for Adverse Events grade 3/4) ranged from 1% in early untreated breast cancer up to 24% in heavily pretreated metastatic breast cancer; grade 4 PN was rare (≤1%). Common symptoms included numbness, paresthesias, and sometimes dysesthesias. Cox regression analysis identified only preexisting neuropathy as a risk factor for increased ixabepilone-associated PN. The management of PN has been primarily through dose adjustments (dose delays and/or dose reduction). Patients had resolution of their neuropathy within a median time of 5 to 6 weeks.

**Conclusions:**

PN is a dose-limiting toxicity associated with ixabepilone treatment, is reversible in most patients, and can be managed with dose reduction and delays.

**Electronic supplementary material:**

The online version of this article (doi:10.1007/s00520-012-1384-0) contains supplementary material, which is available to authorized users.

## Introduction

Microtubules play a fundamental role in diverse cellular functions (cell division, growth, motility) through a very complex dynamic process of polymerization and depolymerization. Hence, they have emerged as an important target for anticancer drugs. The success of the treatments with taxanes in breast cancer and in other tumor types has led to the development of new microtubule-stabilizing agents (MTSAs) as antineoplastic drugs [1]. Among them, the epothilones (ixabepilone, BMS-310705, patupilone, epothilone D, and sagopilone) are the furthest along in clinical development [2–4]. Ixabepilone, the first in a new class of antimicrotubule agents, was approved in the USA and eight other countries both as a monotherapy in metastatic or locally advanced breast cancer after failure of an anthracycline (A), a taxane (T), and capecitabine (C), and as a combination with capecitabine for treatment of metastatic or locally advanced breast cancer resistant to an anthracycline and a taxane.

Peripheral neuropathy (PN), caused by morphologic or functional abnormalities in peripheral nerves, is a nonhematological adverse event associated with all MTSA-based chemotherapies; it may be dose limiting and associated with functional impairment [5, 6]. The nature of PN may vary depending on the type of nerve fibers involved: sensory (manifested by paresthesia, numbness and pain in the feet and hands) [7, 8], motor (usually preceded by sensory neuropathy and usually mild with muscle weakness such as foot drop or difficulty in climbing stairs) [9, 10], or autonomic [rare, observed in less than 1% of patients with metastatic breast cancer (MBC)]. The incidence of grade 3/4 sensory PN in breast cancer patients treated with taxanes ranges from 0% to 33% and that of grade 3/4 motor neuropathy varies from 0% to 14% (Supplemental Table 1).

The clinical assessment of neuropathy usually incorporates a careful evaluation of its onset, distribution, severity, and its impact on quality of life. Early diagnosis and management of mild to moderate (≤grade 2) symptoms are important to prevent progression. Neuropathy is graded by subjective complaints of patients and physical examinations by clinicians. The most widely used grading systems are National Cancer Institute Common Toxicity Criteria (NCI CTC), ECOG and WHO criteria (Supplemental Table 2) [11–13].

The mechanism of MTSA-induced PN is unclear. Preclinical models have demonstrated that both ixabepilone and taxanes produce a very similar neurotoxicity profile. Analysis of accumulation of paclitaxel and ixabepilone in the dorsal root ganglia of mice following intravenous (i.v.) compound administrations showed that at equineurotoxic doses, there was ~10-fold more paclitaxel bound to neuronal microtubule than ixabepilone in peripheral neurons (unpublished data). The pathophysiological consequence of the higher deposit of tubulin-bound drug in peripheral neurons by paclitaxel is currently uncertain, but could be responsible for affecting the duration of neuropathic symptoms and time to resolution following treatment cessation. In this report, we present data on the incidence and characteristics of PN induced by ixabepilone treatment and evaluate potential risk factors for its development.

## Methods

The database of phase II/III clinical trials, involving more than 2,000 patients receiving ixabepilone either as monotherapy or in combination with capecitabine, was searched to obtain incidences of PN. The majority of patients with MBC included into this report received ixabepilone either as monotherapy (40 mg/m^2^ i.v. over 3 h Q3w, *n* = 240) in several phase II studies or in combination with capecitabine (1,000 mg/m^2^ PO BID × 14 days Q3w) in two large phase III studies and one phase II study (*n* = 1,026). The remaining patients (*n* = 274) either received alternate ixabepilone doses (32 mg/m^2^ i.v. over 3 h, 50 mg/m^2^ i.v. Q3w infused over 1 or 3 h, or 6 mg/m^2^ daily for 5 days) for MBC (*n* = 74) or received it as treatment for non-small cell lung cancer (*n* = 195). Patients were considered eligible if they did not have baseline PN >grade 1. PN was graded by investigators using the NCI CTC version 2.0 or 3.0, and the worst grade on treatment was reported; assessments were done prior to each cycle and every 4 weeks after completion of treatment until resolution occurred. Assessments included deep tendon reflexes, sensory function, motor strength, and other neurologic findings, including autonomic function. Neuropathy was followed in all patients in the three pivotal studies of ixabepilone (A/T- and C-resistant phase II study 081[14], A/T-resistant phase III study 046 [15], and A/T-pretreated phase III study 048 [16]) beyond the point that treatment was discontinued to assess for reversibility. Improvement of PN was defined as the time from onset of worst grade to a reduction by at least 1 grade, and resolution was defined as time from onset of worst grade neuropathy to grade 1 or baseline level. Median time to resolution of PN was estimated using the Kaplan–Meier product limit method.

A Cox regression analysis was performed on a dataset of 1,540 patients to identify the potential risk factors for grade 3/4 PN. This analysis included patients with MBC and lung cancer (one study). Patients with MBC were treated with either combination therapy (with capecitabine) or ixabepilone monotherapy in multiple clinical studies across different dose schedules.

## Results

### Incidence

PN, predominantly sensory, has been consistently observed across clinical studies of ixabepilone (phases I–III) in patients with early and metastatic disease (Table 1) [14–22]. In the neoadjuvant setting, the rate for all grades of peripheral sensory neuropathy was 15%, when administered for 4 cycles [17]. Across the three monotherapy studies in MBC [14, 21, 22], incidence of sensory neuropathy (all grades) was 64%; motor neuropathy was less common (all grades, 7%) and was usually reported in patients with peripheral sensory neuropathy (15 of 17 patients with motor neuropathy also reported sensory neuropathy). Painful neuropathy (neuropathic pain, dysesthesia, or neuralgia) was reported in 6% of patients. Incidences of grade 3/4 sensory PN in monotherapy studies ranged from 0% in taxane-naive patients to 14% in taxane-, anthracyclines-, and capecitabine-resistant patients depending on dose, schedule, and setting of administration. In general, grade 4 PN was rare (1%). Grade 3 PN was reported in 20% of patients previously treated with anthracycline.

**Table 1:** Peripheral neuropathy in ixabepilone-treated patients with MBC

Population	Prior MBC Tx	Ref	Ixabepilone (i.v.) dose/schedule	*N*	Median cycles	Incidence of Neuropathy					
						All grades, %		Grade 3, %		Grade 4, %	
						Sensory	Motor	Sensory	Motor	Sensory	Motor
Neoadjuvant phase II											
Untreated	0	[17]	40 mg/m^2^ day 1 Q3w	164	4	20	NR	1	NR	0	NR
Metastatic phase I/II											
A/T pretreated	0–3	[18]	40 mg/m^2^ day 1 Q3w + capecitabine^a^	62	4	74	2	19	2	0	0
Metastatic phase II											
T naive	Any	[19]	6 mg/m^2^ days 1–5 Q3w	23	8	52	8	0	4	0	0
A pretreated	0	[21]	40 mg/m^2^ day 1 Q3w	65	6	71	6	20	5	0	0
T pretreated	0–9	[20]	6 mg/m^2^ days 1–5 Q3w	37		54	0	3	0	0	0
A pretreated/T resistant	1–4	[22]	40 mg/m^2^ day 1 Q3w	49	3	63	2	12	0	0	0
A/T/C resistant	1–3	[14]	40 mg/m^2^ day 1 Q3w	126	4	60	10	13	<1	1	0
Metastatic phase III											
A pretreated/T resistant	0–3	[15]	40 mg/m^2^ day 1 Q3w + capecitabine^b^	369	5	67	16	20	5	<1	0
			Capecitabine^c^	368	4	16	<1	0	0	0	0
A/T pretreated	0–2	[16]	40 mg/m^2^ day 1 Q3w + capecitabine^b^	595	6	66		22	4	1	0
			Capecitabine^c^	603	5	21		1	0	<1	0

*A* anthracycline, *C* capecitabine, *i.v.* intravenous, *NR* not reported, *MBC* metastatic breast cancer, *Q3w* every 3 weeks, *T* taxane, *Tx* treatment lines

^a^Capecitabine 1,650 mg/m^2^ or 2,000 mg/m^2^ days 1–14 PO 3-week cycle

^b^Capecitabine 2,000 mg/m^2^ days 1–14 PO 3-week cycle

^c^Capecitabine 2,500 mg/m^2^ days 1–14 PO 3-week cycle as monotherapy

In trials where ixabepilone was administered in combination with capecitabine, the incidence of PN was common, generally grade 1/2, and reversible. In study 046, 67% of patients had PN, with grades 3 and 4 sensory symptoms reported in 21% and <1% of patients, respectively. Incidence of painful neuropathy was 6%. Severe (grade 3) motor neuropathy was reported in 5% of patients, and no grade 4 event was reported [15]. In study 048, 66% of patients in the combination group had treatment-related PN (24% with grade 3/4), 65% had sensory neuropathy (22% grade 3 and 1% grade 4), and 9% had motor neuropathy (4% with grade 3) [16].

### Factors affecting neuropathy

#### Risk factors

A risk factor analysis was done investigating the association of various covariates [patient age (<65, ≥65), prior taxane therapy, prior other neurotoxic chemotherapy, baseline neuropathy, diabetes, dose levels] with cumulative dose to onset of grade 3/4 PN for 1,540 ixabepilone-treated patients across several clinical studies. In this analysis, the majority of these covariates (age, dose levels, and prior therapy) were not significantly associated with the development of grade 3/4 neuropathy. Prior taxane was paradoxically associated with a decreased risk of severe neuropathy, maybe because of a selection bias where patients who had persistent grade 2 or higher neuropathy from prior therapy were excluded. However, the analysis showed that preexisting neuropathy significantly correlated with a greater risk of grade 3/4 neuropathy (*P* = 0.007, Table 2). This finding is consistent with the individual results from 046 and 048 studies: the rates of grade 3/4 PN for patients with and without baseline neuropathy in 046 were 27% and 22%, respectively, and in 048, were 32% and 22%, respectively. In an earlier analysis that included 945 patients, diabetes mellitus was found to be a significant risk factor [23]; however, this analysis did not identify diabetes as a significant risk factor. Additional analysis (including baseline liver function as a risk factor) showed no influence of baseline hepatic dysfunction on the development of PN. However, since subjects with grade 2 or higher levels of alanine aminotransferase, aspartate aminotransferase, or bilirubin at baseline were excluded from the trials, impact of baseline hepatic impairment on PN cannot be fully evaluated.

**Table 2:** Peripheral neuropathy risk factor analysis—cumulative dose to grade 3/4 neuropathy

	Grade 3/4 neuropathy, % (events/patients)	Median cumulative dose (mg/m^2^)	Hazard ratio	Cox regression, *P* value
Baseline neuropathy				
>Grade 0	22.8 (77/338)	360.3	1.44	0.007
Grade 0	19.5 (234/1,202)	501.0		
Diabetes				
Yes	22.3 (25/112)	Not estimable	1.21	0.375
No	20.0 (286/1,428)	477.9		
Age				
≥65	18.3 (42/229)	561.3	1.19	0.318
17–65	20.5 (269/1,311)	477.9		
Prior taxane				
Yes	20.0 (276/1,379)	543.8	0.35	0.018
No	21.7 (35/161)	306.9		
Prior other chemotherapy				
Yes	16.2 (49/303)	Not estimable	0.75	0.119
No	21.2 (262/1,237)	441.3		

The risk factor analysis was conducted on ixabepilone-treated patients from studies CA163009 (monotherapy, taxane resistant) [22], CA163010 (monotherapy, anthracycline pretreated) [21], CA163011 (monotherapy, platinum-pretreated non-small cell lung cancer) [24], CA163031 (combination with capecitabine, anthracycline, and taxane pretreated) [18], CA163046 (combination with capecitabine, anthracycline, and taxane resistant) [15], CA163048 (combination with capecitabine, anthracycline, and taxane pretreated) [16], and CA163081 (monotherapy, taxane, and capecitabine resistant) [14]. Additional factors, that were included in the regression analysis, but not presented in the table includes dose schedules of 40 mg/m^2^ 3 h, 32 mg/m^2^ 3 h, 50 mg/m^2^ 1 h, 50 mg/m^2^ 3 h, and 6 mg/m^2^ days 1–5. These factors were not statistically significant

#### Dose per cycle

The incidence of PN is also correlated with the dose of ixabepilone administered per treatment cycle and the duration of infusion. In several clinical studies where ixabepilone was given at a dose of 40 mg/m^2^ every 3 weeks infused over 3 h, grade 3/4 PN was observed in 15% to 24% of patients [14–16, 21, 22]. In clinical trials where a higher dose (50 mg/m^2^) of ixabepilone was infused for 1 h, 17% to 38% of patients showed evidence of severe neuropathy; when infused for 3 h, 11% to 33% of patients had severe PN [21]. In a clinical trial testing with a lower dose of 32 mg/m^2^ infused for 3 h, the incidence of grade 3/4 PN was reduced (5% of patients had grade 3/4 PN) [24]. Lower incidences of PN (3%) were also observed when ixabepilone was administered at 6 mg/m^2^/day on days 1 through 5 of a 3-week cycle [20].

#### Cumulative dose

**Fig. 1:**
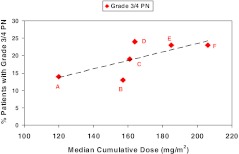
Grade 3/4 peripheral neuropathy rate increases with increase in median cumulative dose of ixabepilone administered to patients in five breast cancer studies. *A* T-resistant MBC [22], *B* A/T/C-resistant MBC [14], *C* A-pretreated/resistant MBC [18], *D* A/T-pretreated MBC [16], *E* A-pretreated/T-resistant MBC [15], *F* A-pretreated MBC [21]

Ixabepilone-associated PN appears to be cumulative. In phase II clinical studies where ixabepilone was administered at 40 mg/m^2^, 3% to 20% of patients at various stages of tumor progression developed grade 3/4 PN, after receiving a median of 3 to 6 treatment cycles. The incidence of grade 3/4 PN increased with the median cumulative dose received in the different clinical studies (Fig. 1): incidence was 12% at 120 mg/m^2^ (range 39.6–489.8 mg/m^2^) [22], 14% at 156.9 mg/m^2^ (range 0.6–626.3 mg/m^2^) [14], 19% at 161.1 mg/m^2^ (range 39.1–799.6 mg/m^2^) [18], 21% at 184.9 mg/m^2^ (range 37.6–719.2 mg/m^2^) [15], and 23% at 206.5 mg/m^2^ (range 40.1–466.1 mg/m^2^) [21]. These treatment cycles were sufficient for the patients to achieve optimal efficacy. The median cycles received in patients with PN were in line with the median cycles of treatment received by all treated patients in these trials. Therefore, the patients with PN could receive sufficient cycles of ixabepilone to achieve efficacy. In study 081, severe (grade 3/4) PN developed in 17 of 126 patients (13%) after a median of 4 cycles (range 1–11 cycles); the time to the onset of grade ≥3 was found to be gradual, with a low probability of occurrence at 3 months [14].

In both the phase III studies, some occurrences of severe PN were reported in earlier cycles, but the proportion of patients with extensive symptoms generally increased with additional cycles of treatment, reinforcing the cumulative nature of the toxicity. In study 046, 79 of 369 patients (21%) developed severe grade 3/4 treatment-related PN in the combination arm [15]. The median time to onset among patients with grade 3/4 PN was 2.9 months (approximately 4 cycles). In addition, based on a Kaplan–Meier analysis including all treated patients (combination, *n* = 369), the median time to onset was 13.3 months (Table 3, Supplemental Fig. 1). Also, among these 79 patients, the median cumulative dose to onset of grade 3/4 PN was 163 mg/m^2^. For this analysis, patients who did not develop severe PN were censored at 30 days after the last dose. Of the 79 patients treated with ixabepilone combination who developed grade 3/4 PN, 39 developed symptoms after more than 4 cycles. Kaplan–Meier analysis of cumulative dose to grade 3/4 PN showed that the probability was 13% for patients who received at least 4 cycles of ixabepilone (approximately the median number of cycles needed to achieve an objective response, 12 weeks = 4 cycles).

**Table 3:** Time to onset of grade 3/4 peripheral neuropathy computed from the ixabepilone + capecitabine arms of phase III trials

Study [reference]	046 [15]	048 [16]
Number of patients	369	595
Grade 3/4 PN, *n* (%)	79 (21)	140 (24)
Time (months, 95% CI), among patients with grade 3/4 PN	2.9 (2.4–3.4)	3.0 (2.7–3.6)
Time (months, 95% CI), among all treated patients^a^	13.3 (9.3–15.5)	(10.3–NR)^b^

*NR* not reached, *PN* peripheral neuropathy

^a^Patients who did not experience grade 3/4 PN were censored 30 days after last dose of ixabepilone

^b^Median was not reached for all treated patients

Similarly, in study 048, 144 patients developed grade 3/4 PN; half of these patients (69 of 140) did so after 4 cycles. Among those patients in the combination group who developed grade 3/4 PN, the median cumulative dose to onset was 164 mg/m^2^ and the median time to onset was 3 months (approximately 4 cycles). The probability of developing grade 3/4 PN was 14% for patients who received at least 4 cycles of ixabepilone [16].

### Management

#### Resolution of neuropathy

In the pivotal studies where ixabepilone was either used as monotherapy [14], or in combination with capecitabine [15], PN was found to be reversible (Table 4). In study 081, 13 of the 17 patients with grade 3/4 neuropathy had documented resolution to baseline or grade 1 in a median time of 5.4 weeks and 14 had an improvement in a median time of 4.6 weeks; 47 patients with grade ≥2 PN had documented resolution in a median time of 4.0 weeks [14]. Among the four patients lacking documented resolution, two patients received subsequent neurotoxic therapy, one patient had no follow-up treatment, and one patient improved to grade 2 after 4 weeks (but with no additional improvement during the ensuing 7 weeks of follow-up).

**Table 4:** Time to resolution and improvement of grade 3/4 peripheral neuropathy

	Ixabepilone	Ixabepilone + capecitabine	
Study [reference]	081 [14]	046 [15]	048 [16]
Patient number	126	369	595
Grade 3/4 PN, *n* (%)	17 (14)	79 (21)	140 (24)
Resolution			
Resolution^a^, *n* (%)	13 (76)	70 (89)	120 (86)
Median time to resolution, weeks (95% CI)	5.4 (3.3–11.4)	6.0 (4.6–7.6)	6.2 (5.0–8.7)
Improvement			
Improvement, *n* (%)	14 (82)	70 (89)	124 (89)
Median time to improvement, weeks (95% CI)	4.6 (0.9–6.1)	4.1 (2.9–6.0)	4.5 (3.3–5.1)

^a^Patients who did not have resolution of PN were censored due to death, subsequent neurotoxic treatment, and lost to follow-up

In study 046, 70 of the 79 patients (89%) with treatment-related grade 3/4 PN had documented resolution of their symptoms in a median time of 6.0 weeks. All three patients with treatment-related grade 4 sensory neuropathy achieved resolution of PN (in 1 to 5 months) [15]. These patients had improvement of their symptoms in a median time of 4.1 weeks. Nine patients lacking documented resolution (or improvement) were censored at the time of the analysis due to receiving subsequent neurotoxic treatment (four patients), death (two patients), lost to follow-up (two patients), and continuing on treatment after onset of neuropathy (one patient). Resolution of PN in patients with grade ≥2 neuropathy was consistent with these results; 163 out of 183 patients had documented resolution, and the median time to resolution was 6.0 weeks. Improvement was noted in 163 patients in a median time of 5.1 weeks.

In study 048, 120 of the 140 patients (86%) with treatment-related grade 3/4 PN had resolution to grade 1 or baseline in a median time of 6.2 weeks from the onset of the severe neuropathy. The median time to improvement of grade ≥3 treatment-related neuropathy by at least 1 grade was 4.5 weeks. Twenty patients lacked documented resolution at the time of the analysis of which 4 had an improvement by 1 grade, 10 patients were dead, 4 patients went on to receive subsequent neurotoxic therapy, and 2 patients were still on treatment at the time of the analysis.

#### Dose reduction and delay

PN associated with ixabepilone treatment was effectively managed by dose reductions or delay (Table 4). In study 081, 23 of the 33 patients (70%) with grade 2 PN lasting ≥7 days or grade 3/4 PN received a dose reduction [14]. They received a median of three additional cycles after the dose reduction; 20 of them (87%) had improvement or no worsening of their neuropathy.

Similarly, in study 046, 116 (32%) patients with grade 2 PN lasting ≥7 days or with grade 3/4 PN received further treatment with ixabepilone [15]. Of these, 84 patients (72%) with persistent grade 2/3 neuropathy qualified for dose reduction; they received a median of three additional cycles (range 1–16) at the reduced dose. Sixty seven of these 84 patients (80%) reported improvement or no worsening of their neuropathy following dose reduction. In study 048, 162 (27%) patients with persistent grade 2/3 neuropathy were eligible for dose reduction. Of these, 115 (71%) had dose reduction and received a median of three additional cycles (range 1–32); 86 (75%) had improvement or no worsening of their neuropathy [16].

To assess the impact of dose reductions on the overall efficacy, a retrospective analysis of progression-free survival (PFS) and overall survival (OS) was conducted in patients treated with ixabepilone plus capecitabine in the two phase III trials with early dose reductions (dose reduction within the first four courses) relative to those with no early dose reductions (dose reduction after the first four courses or no dose reductions) [25]. The results indicated that the efficacy (both PFS and OS) was similar in both these groups [hazard ratio (HR) of no early dose reduction over early dose reduction: PFS HR = 1.15, 95% CI = 0.86–1.53, OS HR = 1.13, 95% CI = 0.85–1.50]. To adjust for the bias resulting from selecting patients in one group who may have an outcome inherently better than the other based on the duration of therapy received, these analyses were restricted to those patients who received at least four courses of ixabepilone treatment.

## Conclusions

PN is the predominant side effect of ixabepilone similar to other tubulin-targeting agents, as well as other anticancer agents with different mechanisms of action. PN associated with ixabepilone is primarily sensory and cumulative. The median time from onset to resolution (return to baseline or grade 1 severity) is 5 to 6 weeks in patients who develop severe neuropathy. Data from the phase II studies indicated that the incidence of PN is correlated with the dose of ixabepilone administered per treatment cycle, the duration of infusion, and the cumulative dose. A regression analysis evaluating the association of several prognostic factors with PN found preexisting neuropathy to be significantly associated with onset of grade 3/4 PN. None of the other factors tested in the analyses appeared to be associated with an increased risk of severe neuropathy from ixabepilone. In an earlier analysis that included 945 patients, diabetes mellitus was found to be a significant risk factor, which was not observed in this analysis.

In all trials of patients with heavily pretreated MBC and other advanced solid tumors such as refractory prostate cancer, neuropathy has been managed with dose reduction and treatment delay [14–16, 18–22, 26, 27]. No study with a neuroprotectant has been conducted. Many patients have been able to continue therapy after the ixabepilone dose was reduced [14–16, 26, 27]. Not much information is available to compare the time course of this reversibility with resolution of sensory symptoms of other MTSA-related PN; taxane-induced PN also improved after completion or discontinuation of therapy, although specific data on the time course of this process are infrequently reported [28, 29]. In many cases, there is no report of resolution to baseline. Also, the PN observed with this drug is different in that dysesthesias may be observed and over the course of 1 cycle, symptoms can progress from mild to severe (data not shown). Therefore, it is prudent to institute a dose reduction or delay at the first sign of moderate numbness and paresthesias [30]. In conclusion, PN is a dose-limiting toxicity associated with ixabepilone treatment, which is reversible in the majority of patients and can be managed fairly easily with dose reduction and delays.

## Electronic supplementary material

Below is the link to the electronic supplementary material.

Supplemental Table 1 (DOCX 24.9 kb)

Supplemental Table 2 (DOCX 36 kb)

Supplemental Fig. 1 (DOCX 123 kb)
