# Supplementary material for: Ixabepilone-associated peripheral neuropathy: data from across the phase II and III clinical trials
Source: Support Care Cancer. 2012 Mar 2;20(11):2661–8. doi: 10.1007/s00520-012-1384-0 (PMC3461204; doi:10.1007/s00520-012-1384-0)
Supplement: Supplementary file 1 — (DOCX 24.9 kb) [file 520_2012_1384_MOESM1_ESM.docx]

Supplemental Table 1

Incidence of peripheral neuropathy in breast cancer patients treated with taxanes

| Drug/schedule  (i.v.) | Dose (mg/m^2^) | Patient number | Grade 3/4 neuropathy | | Reference |
| --- | --- | --- | --- | --- | --- |
|  |  |  | Sensory (%) | Motor (%) |  |
| Paclitaxel |  |  |  |  |  |
| 1h every week | 80 | 228 | 19 | 7 | Seidman [31] |
|  |  | 212 | 9 | 0 | Perez [32] |
| 3h Q3w | 175 | 150 | 7 | 5 | Winer [33] |
|  |  | 225 | 2 | 0 | Gradishar [29] |
|  |  | 222 | 5 | 5 | Jones [34] |
|  |  | 224 | 12 | 4 | Seidman [31] |
|  | 200 | 164 | 9 |  | Paridaens [35] |
|  | 210 | 152 | 19 | 11 | Winer [33] |
|  | 250 | 149 | 33 | 13 | Winer [33] |
|  |  | 278 | 13 | 9 | Smith [36] |
| 24h Q3w | 250 | 279 | 7 | 7 | Smith [36] |
| Docetaxel |  |  |  |  |  |
| 1h Q3w | 60 | 150 | 1 |  | Harvey [37] |
|  | 75 | 188 | 2 |  | Harvey [37] |
|  | 100 | 186 | 4 |  | Harvey [37] |
|  |  | 159 | 5 | 5 | Chan [38] |
|  |  | 200 | 5 |  | Nabholtz [39] |
|  |  | 222 | 9 | 9 | Jones [34] |
|  |  | 253 | 2 | 1 | Kruijtzer [40] |
| Paclitaxel, albumin-bound (ABI-007) |  |  |  |  |  |
| 30 min every week | 100 | 106 | 8 |  | Blum [41] |
|  | 125 | 75 | 19 |  | Blum [41] |
| 30 min Q3w | 175 | 43 | 0 |  | Ibrahim [42] |
|  | 260 | 229 | 11 | 0 | Gradishar [29] |
|  | 300 | 63 | 11 |  | Ibrahim [42] |

*i.v.* intravenous*; Q3w* every 3 weeks

References

31. Seidman AD, Berry D, Cirrincione C, et al (2008) Randomized phase III trial of weekly compared with every-3-weeks paclitaxel for metastatic breast cancer, with trastuzumab for all HER-2 overexpressors and random assignment to trastuzumab or not in HER-2 nonoverexpressors: final results of Cancer and Leukemia Group B protocol 9840. J Clin Oncol 26:1642–1649

32. Perez EA, Vogel CL, Irwin DH, et al (2001) Multicenter phase II trial of weekly paclitaxel in women with metastatic breast cancer. J Clin Oncol 19:4216–4223

33. Winer EP, Berry DA, Woolf S, et al (2004) Failure of higher-dose paclitaxel to improve outcome in patients with metastatic breast cancer: cancer and leukemia group B trial 9342. J Clin Oncol 22:2061–2068

34. Jones SE, Erban J, Overmoyer B, et al (2005) Randomized phase III study of docetaxel compared with paclitaxel in metastatic breast cancer. J Clin Oncol 23:5542–5551

35. Paridaens R, Biganzoli L, Bruning P, et al (2000) Paclitaxel versus doxorubicin as first-line single-agent chemotherapy for metastatic breast cancer: a European Organization for Research and Treatment of Cancer Randomized Study with cross-over. J Clin Oncol 18:724–733

36. Smith RE, Brown AM, Mamounas EP, et al (1999) Randomized trial of 3- hour versus 24-hour infusion of high-dose paclitaxel in patients with metastatic or locally advanced breast cancer: National Surgical Adjuvant Breast and Bowel Project Protocol B-26. J Clin Oncol 17:3403–3411

37. Harvey V, Mouridsen H, Semiglazov V, et al (2006) Phase III trial comparing three doses of docetaxel for second-line treatment of advanced breast cancer. J Clin Oncol 24:4963-4970

38. Chan S, Friedrichs K, Noel D, et al (1999) Prospective randomized trial of docetaxel versus doxorubicin in patients with metastatic breast cancer. J Clin Oncol 17:2341–2354

39. Nabholtz JM, Gelmon K, Bontenbal M, et al (1996) Multicenter, randomized comparative study of two doses of paclitaxel in patients with metastatic breast cancer. J Clin Oncol 14:1858–1867

40. Kruijtzer CMF, Verweij J, Schellens JH, et al (2000) Docetaxel in 253 previously treated patients with progressive locally advanced or metastatic breast cancer: results of a compassionate use program in The Netherlands. Anticancer Drugs 11:249–255

41. Blum JL, Savin MA, Edelman G, et al (2007) Phase II study of weekly albumin-bound paclitaxel for patients with metastatic breast cancer heavily pretreated with taxanes. Clin Breast Cancer 7:850-856

42. Ibrahim NK, Samuels B, Page R, et al (2005) Multicenter phase II trial of ABI-007, an albumin-bound paclitaxel, in women with metastatic breast cancer. J Clin Oncol 23:6019–6026
