# Supplementary material for: Ixabepilone-associated peripheral neuropathy: data from across the phase II and III clinical trials
Source: Support Care Cancer. 2012 Mar 2;20(11):2661–8. doi: 10.1007/s00520-012-1384-0 (PMC3461204; doi:10.1007/s00520-012-1384-0)
Supplement: Supplementary file 2 — (DOCX 36 kb) [file 520_2012_1384_MOESM2_ESM.docx]

Supplemental Table 2

Grading systems for peripheral neuropathy

| Scale | Grade 0 | Grade 1 | Grade 2 | Grade 3 | Grade 4 |
| --- | --- | --- | --- | --- | --- |
| WHO | None | Paresthesias and/or decreased deep tendon reflexes | Severe paresthesias and /or mild weakness | Intolerable paresthesias and/or motor loss | Paralysis |
| ECOG | None | Decreased deep tendon reflexes, mild paresthesias, mild constipation | Absent DTR, severe constipation, mild weakness | Disabling sensory loss, severe peripheral neuropathic pain, obstipation, severe weakness, bladder dysfunction | Respiratory dysfunction secondary to weakness, obstipation requiring surgery, paralysis confining patient to bed/wheelchair |
| NCI CTC  sensory | None | Loss of deep tendon reflexes or paresthesia (including tingling) but not interfering with function | Objective sensory loss or paresthesia interfering with function, but not interfering with activities of daily living | Sensory loss or paresthesia interfering with activities of daily living | Permanent sensory loss that interferes with function |
| NCT CTC motor | None | Subjective weakness but no objective findings | Mild objective weakness interfering with function, but not interfering with activities of daily living | Objective weakness interfering with activities of daily living | Paralysis |
