# Supplementary material for: Ixabepilone-associated peripheral neuropathy: data from across the phase II and III clinical trials
Source: Support Care Cancer. 2012 Mar 2;20(11):2661–8. doi: 10.1007/s00520-012-1384-0 (PMC3461204; doi:10.1007/s00520-012-1384-0)
Supplement: Supplementary file 3 — (DOCX 123 kb) [file 520_2012_1384_MOESM3_ESM.docx]

Supplemental Figure 1

Time to onset of grade 3/4 peripheral neuropathy (worst grade)
